# Local Excision of a Mucinous Adenocarcinoma of the Anal Margin (Extramammary Paget's Disease) and Reconstruction with a Bilateral V-Y Flap

**DOI:** 10.1155/2019/9073982

**Published:** 2019-12-03

**Authors:** Michele Pagnanelli, Paola De Nardi, Stefano Martella, Luca Caruso, Riccardo Rosati

**Affiliations:** ^1^Gastrointestinal Surgery, San Raffaele Scientific Institute, Milan 20132, Italy; ^2^Senology and Reconstructive Surgery, Policlinico Abano Terme, Abano Terme 25031, Italy

## Abstract

The case of a 75-year-old female with invasive extramammary Paget's disease of the anal margin, without involvement of the anal canal, is reported. The patient underwent wide local excision of the lesion with reconstruction with a double V-Y flap, a biopsy of the inguinal sentinel node, and a laparoscopic temporary colostomy. No guidelines exist on the treatment of this rare disease, and both wide local excision and abdominoperineal resection have been proposed. In the present case, the absence of invasion of the anal canal, also confirmed by intraoperative biopsies on the resection margins, and of local lymph node metastasis, as confirmed by the sentinel lymph node biopsy, allowed a sphincter-sparing operation with good functional and oncological results.

## 1. Introduction

Mucinous adenocarcinoma of the perianal skin is an extremely rare disease [1]. It usually arises after long-lasting untreated Paget's disease [2]. The treatment often requires an abdominoperineal excision; however, in the absence of regional nodes and anal canal involvement, a wide local excision can be carried out to avoid a permanent colostomy. Large resections may require various flaps to close the surgical defect and the assistance of a plastic surgeon [3].

We present a case of a local excision of a mucinous adenocarcinoma of the anal margin and reconstruction with a bilateral V-Y flap.

## 2. Case Presentation

**Figure 1:**
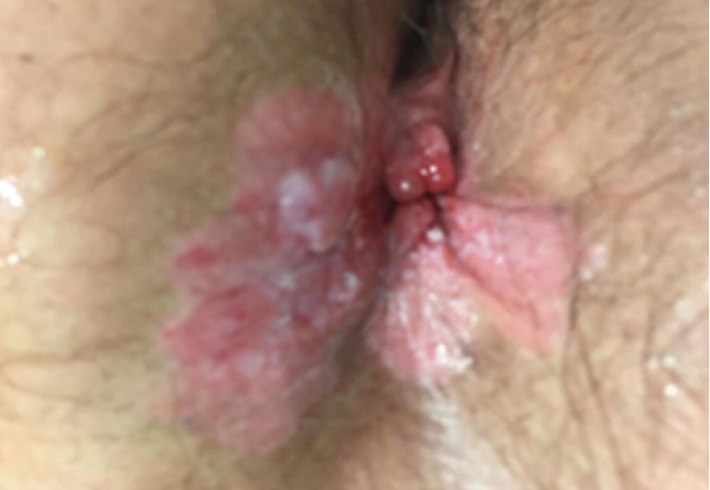
Preoperative appearance of the mucinous adenocarcinoma.

**Figure 2:**
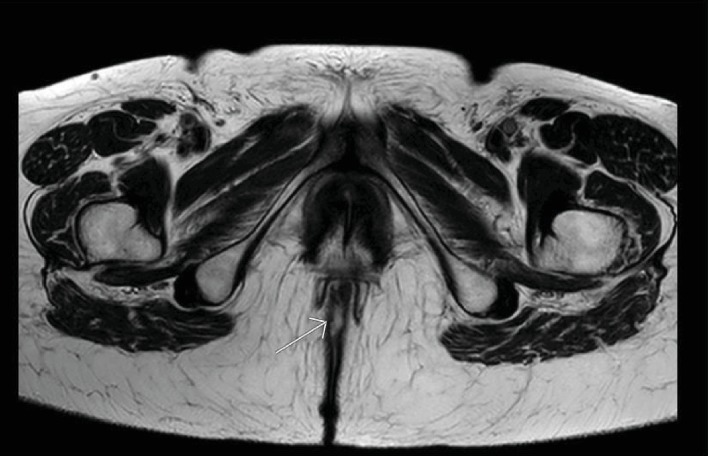
Pelvic MRI (T2W-TSE) showing invasion of the skin but not of the anal sphincters.

A 75-year-old female patient presented at the surgical consultation for perianal hitching and bleeding. The patient's complaints started 12 months prior, with progressive worsening. Her past medical history included hypothyroidism, glaucoma, and hemorrhoidectomy 30 years previously. Her family medical history included hepatic cirrhosis (father) and coronary artery disease (mother) but no cancer. Physical examination showed a 3 cm plaque-like eczematous lesion, circumferentially involving the perianal skin and extending to the anal verge (Figure 1). A biopsy of the perianal mass demonstrated a well-differentiated mucinous adenocarcinoma. Serum carcinoembryonic antigen level and other laboratory tests were within normal range. A pelvic MRI showed that the lesion had invaded the skin tissue without involvement of perirectal structures and anal sphincters (Figure 2). An endoanal ultrasound confirmed the absence of invasion into the anal canal and anal sphincters. A colonoscopy showed two polyps in the ascending colon that were both removed and histologically examined; they were both tubular-villous adenomas with low-grade dysplasia. A total-body CT scan did not show any associated visceral malignancy or lymphadenopathy. The patient was discussed within a multidisciplinary team including surgeons, oncologists, radiotherapists, radiologists, and pathologists. Since no anal canal involvement was identified and because of the patient's refusal of a permanent colostomy, a wide local excision was planned with a flap to close the perianal defect, and a protective colostomy. A biopsy of the inguinal sentinel node was also scheduled to rule out inguinal lymph node metastasis.

**Figure 3:**
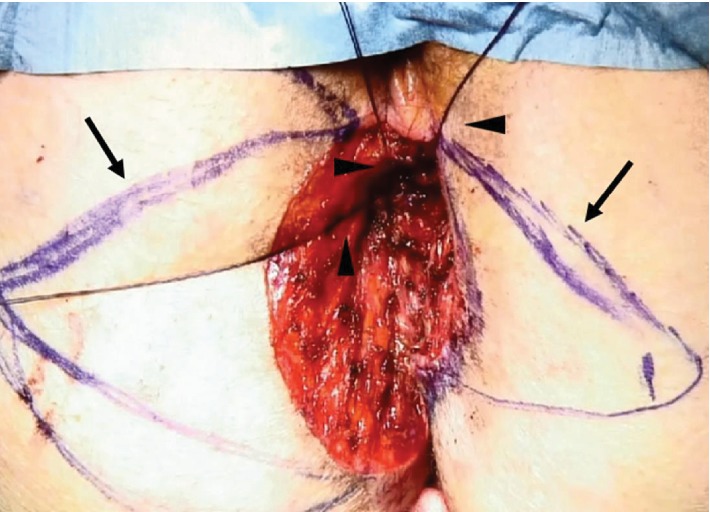
Intraoperative surgical field after resection of the lesion with the double V flap (arrows) marking drawn and the stiches placed on the margins of the anal mucosa (arrowheads).

The day before the operation, a lymphoscintigraphy was performed which showed uptake in the right inguinal region. The patient underwent surgery the following day under general anesthesia. A sentinel inguinal lymph node was retrieved at the right groin region. A laparoscopic lateral colostomy was performed on the sigmoid colon. The patient was then placed in a jackknife position. The neoplasia was resected with 1 cm of free margins over the skin and the anal verge, including the dermis and the subcutaneous tissue. Once the excision was completed, multiple biopsies inside the anal canal were taken to exclude invasion. Then, stiches were placed at the four cardinal points on the mucosa of the anal canal. A double V marking was drawn, and the flaps were dissected with vascular pedicle preservation, based on random inferior gluteal perforator vessels and peripheral skin undermining (Figure 3). The flaps were extended medially until complete coverage of the defect was attained and were then sutured tension free to the mucosa of the anal canal. The subcutaneous tissue was approximated, and the dermis was closed with separate sutures of reabsorbable material.

**Figure 4:**
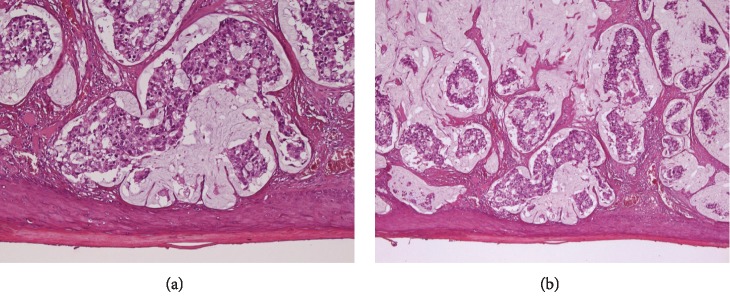
(a, b) Histology of the perianal lesion showing a discontinuous spread of pagetoid cells (EE: 20x (a); 5x (b)).

**Figure 5:**
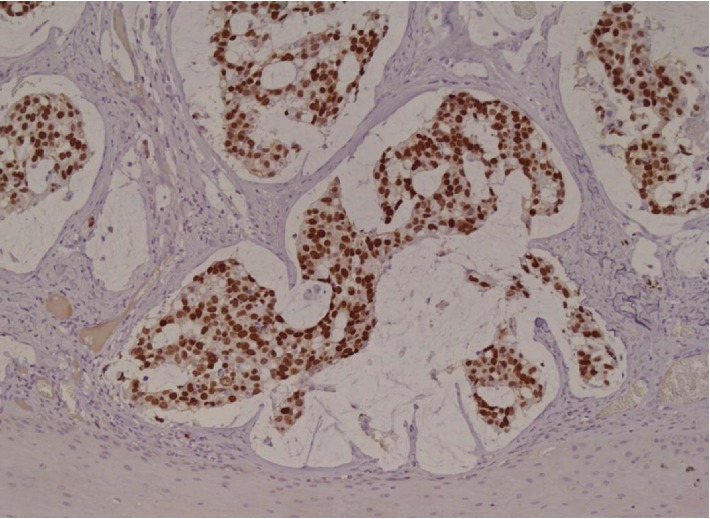
Immunohistochemistry showing positivity for CDX2, typical of secondary (intestinal) adenocarcinoma (20x).

**Figure 6:**
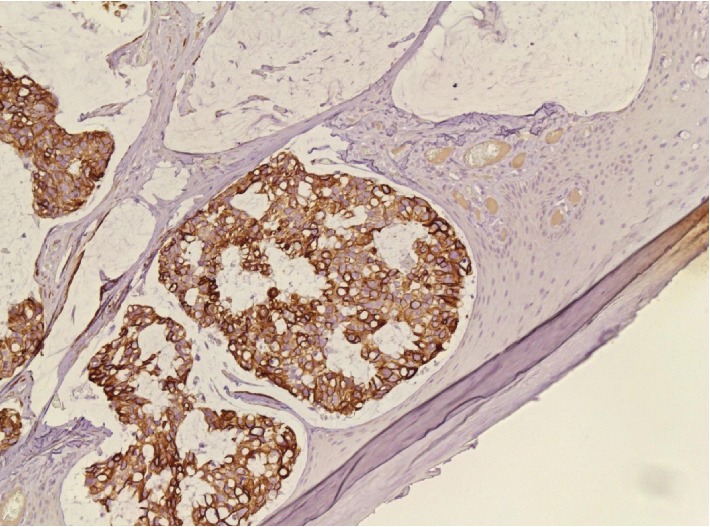
Immunohistochemistry showing positivity for CK7 (20x).

**Figure 7:**
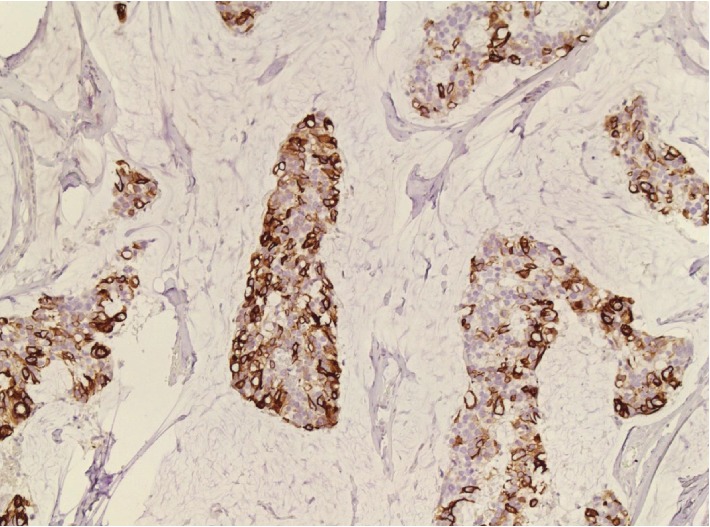
Immunohistochemistry showing positivity for CK20 (20x).

**Figure 8:**
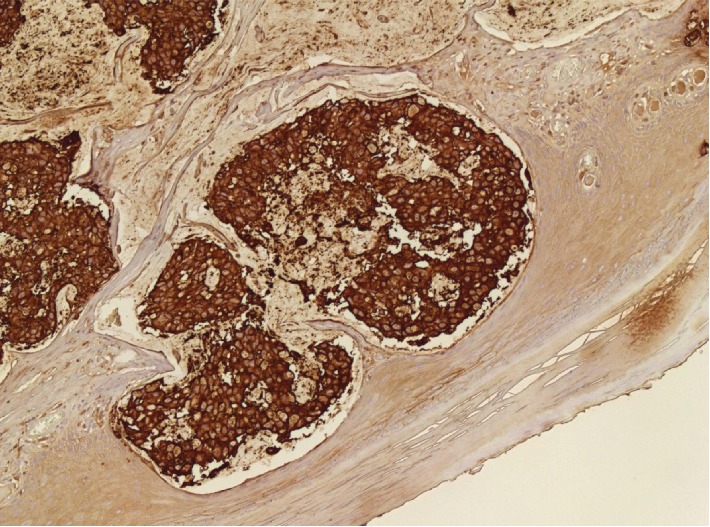
Immunohistochemistry showing positivity for CEA (20x).

**Figure 9:**
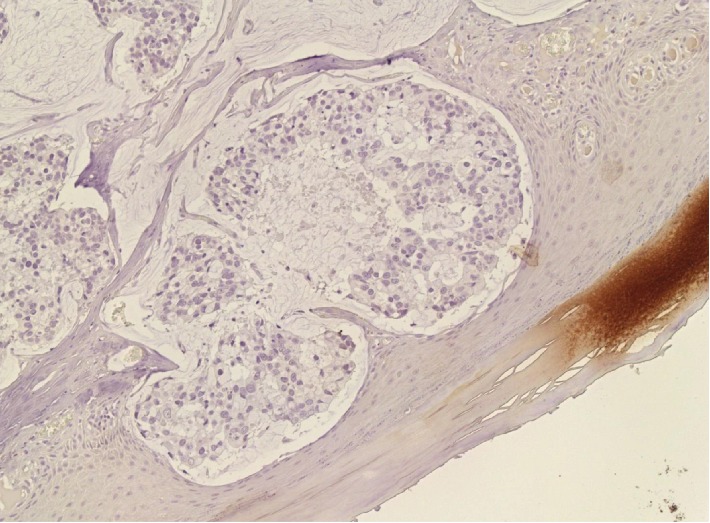
Immunohistochemistry showing negativity for GCFDP15 (20x).

The pathological report was mucinous adenocarcinoma of the perianal skin involving the subcutaneous tissue with pagetoid spread with negative resection margins (Figure 4). The lesion presented the following immunohistochemical pattern: CK7+, CK20+, CDX2+, CEA+, and GCFDP15-, suggestive of secondary Paget's disease (Figures 5–9). No metastasis on the inguinal sentinel node was demonstrated. The postoperative course was uneventful, and the patient was instructed to avoid recumbent or sitting position for the first 4 days and was discharged on postoperative day 12.

**Figure 10:**
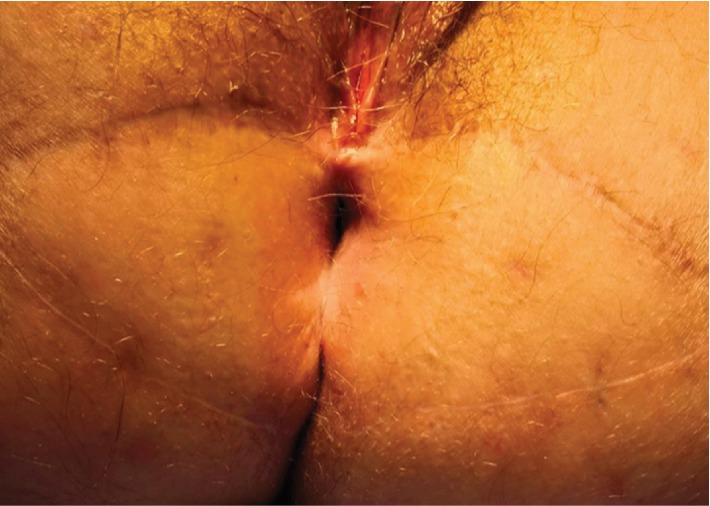
View of the perianal region three months after surgery.

After 3 months (Figure 10), random biopsies were taken in the flaps, in the skin proximal to the flaps, and in the anal canal, and the histopathological examination showed no sign of disease. The colostomy was then closed 2 months later. She was followed up at 3-month intervals; at the last follow-up, 14 months after surgery, the patient was fully continent and did not show any sign of recurrence.

## 3. Discussion

Perianal or anal margin cancers are defined as cancers that arise distally from the dentate line and can be entirely seen by gentle traction to the buttocks [4]; they are rare diseases accounting for 3-4% of all anorectal malignancies [2]. Adenocarcinomas of the perianal region are extremely rare and can originate from anorectal glands or long-standing fistulas extending down from the proximal anal canal or arising primarily from the apocrine glands of the perianal skin [4]. Perianal Paget's disease (PPD) has been believed to be the precursor lesion for perianal adenocarcinoma, with a prolonged preinvasive phase before its eventual growth into invasive cancer. About 40% of PPD is associated with visceral malignancies particularly in the gastrointestinal tract. This latter form is also known as secondary-type Paget's disease (PD) that usually carries a worse prognosis with respect to the primary type [5]. Consequently, for a thorough evaluation of this disease, it is mandatory to utilize a CT scan, a gynecological evaluation, and a colonoscopy in order to exclude the presence of other lesions [6].

Immunohistochemical studies can help differentiate primary from secondary PD since the presence of CK7 and GCDFP15 suggests primary PD while CK20 and CDX2 are more consistent with secondary PD [7, 8]. In the present case, the immunohistochemical pattern indicated a secondary PD. Nevertheless, the colonoscopy did not show any malignancy but instead two benign polyps. To the best of our knowledge, only three cases are reported of perianal Paget's disease in association with adenomatous lesions of the colon without invasive cancer [5, 9, 10].

The management of PPD is not standardized and includes noninvasive approaches (topical imiquimod, chemotherapy, radiotherapy, and photodynamic therapy) as well as surgical treatments (wide local excision or abdominoperineal resection) [2, 11–13]. Invasive PPD usually requires abdominoperineal resection [14]; however, wide local excision (WLE) enables preservation of the anal sphincter, optimizes patients' quality of life, and can be proposed in select cases such as the present case in which no invasion of the anal canal and sphincters was demonstrated. Moreover, the biopsy of the inguinal sentinel lymph node allowed us to refine the locoregional staging, thus excluding the presence of subclinical inguinal metastasis [15, 16]. WLE is usually associated with a wide skin defect that can be closed primarily or with the use of myocutaneous, rotational, or advancement skin flaps. In most situations, especially for lesions that involve more than half of the circumference of the anus or with a radius of more than 3 cm, WLE is associated with temporary fecal diversion in order to prevent wound infection and consequent flap failure [17, 18]. After surgery, a tight follow-up, in order to exclude early disease recurrence, is mandatory. The recurrence rate after WLE is quite high even for noninvasive lesions, as observed by Perez et al. reporting 56% and 29% recurrence rates for invasive and noninvasive lesions, respectively [3]. This is because PPD tends to present a discontinuous spread of pagetoid cells in the epidermis beyond the clinically apparent margin, and the entire lesion is consequently very difficult to remove with no residual cells remaining [19]. As a consequence, according to the literature, after surgery, patients with noninvasive lesions must undergo periodic proctoscopies and digital examinations, while the follow-up of patients with invasive diseases should include CT scan for lung and liver evaluation as well as the examination of inguinal lymph nodes [20]. There are no randomized trials in the literature to demonstrate the effectiveness of adjuvant therapy for this type of disease [6].

## 4. Conclusions

This rare disease can be successfully treated with wide local excision after a throughout preoperative workup and intraoperative exclusion of anal canal involvement, thus avoiding a permanent colostomy. Attention should be paid to exclude concomitant malignancies and to differentiate primary from secondary disease through immunohistochemistry on the resected specimen.
